# Multidimensional Experience of Pain in Adults With Cirrhosis: A Cross-sectional Survey Study

**DOI:** 10.1016/j.gastha.2025.100734

**Published:** 2025-06-24

**Authors:** Franklin F.F. Gorospe, David Wong, Elizabeth Lee, Martine Puts, Sarah Brennenstuhl, Craig M. Dale

**Affiliations:** 1 Lawrence S. Bloomberg Faculty of Nursing, University of Toronto, Toronto, Ontario, Canada; 2 Perioperative Services, Toronto General Hospital, University Health Network, Toronto, Ontario, Canada; 3 Daphne Cockwell School of Nursing, Toronto Metropolitan University, Toronto, Ontario, Canada; 4 Toronto Centre for Liver Disease, Toronto General Hospital, University Health Network, Toronto, Ontario, Canada; 5 Tory Trauma Program, Sunnybrook Health Sciences Centre, Toronto, Ontario, Canada; 6 University of Toronto Centre for the Study of Pain, Toronto, Ontario, Canada

**Keywords:** Pain management, Liver disease, Multidimension, Cirrhosis

## Abstract

**Background and Aims:**

Patients with liver disease report unrelieved pain. Exploring the multidimensional nature of pain provides insights into its burden and management needs in cirrhosis. This study explored biopsychosocial factors of pain and the relationship between pain intensity and liver disease severity.

**Methods:**

A cross-sectional survey design was conducted at a Canadian ambulatory hepatology clinic in 2021. Eligible participants were ≥18 years with a documented the Model for End-stage Liver Disease with Sodium (MELD-Na) score. We report pain characteristics, self-care management, and pain interference using the Brief Pain Inventory. Multiple linear regression analyses examined the relationship between MELD-Na and pain intensity.

**Results:**

Of 118 participants (98% response rate), 61.9% were male, 59 ± 11.8 years of age. Leading diagnoses were alcohol related (34.7%) and viral hepatitis (21.2%), and 89.8% had decompensated cirrhosis. The composite pain intensity mean score was 4 ± 2.4. Common pain sites included: abdomen (83.1%), lower legs (59.3%), and lower back (56.8%). Pain interference scores ranged from general activity 5.8 ± 3.1, walking ability 5.7 ± 3.3, and sleep 5.6 ± 3.6. Use of self-management strategies for pain was reported by 37.3%, primarily oral analgesics; no social or psychological strategies were reported. Multiple regression analysis indicated that pain intensity was associated with liver disease severity. MELD-Na 23–31 (β = 0.43), 21–22 (β = 0.28), and 17–20 (β = 0.19). Physical factors associated with pain intensity included ascites (β = 0.16) and edema (β = 0.28).

**Conclusion:**

We observed moderate pain and pain-related activity interference in adults with cirrhosis. Ascites, edema, and liver disease severity were associated with pain intensity. More research is needed to explore biopsychosocial pain management interventions for this population.

## Introduction

Cirrhosis poses a significant global public health challenge, with 1.47 million deaths worldwide in 2019, a 45.3% increase from 1.01 million in 1990.^1^^,^^2^ In Canada, approximately 10 million individuals may have liver disease that can lead to cirrhosis, reflecting a substantial national burden.^3^ Projections indicate a rising liver disease burden by 2030,^4^ with cirrhosis consistently among Canada’s top 10 causes of death in 2023.^5^ In cirrhosis, the liver is scarred and its function significantly diminishes, preventing it from meeting the body’s metabolic requirements. This can lead to systemic complications and may eventually result in death.^6^, ^7^, ^8^ Patients with cirrhosis are known to have higher health-care use because of a compounding symptom burden including pain.^9^ Comprehensive pain assessment is important for patients with cirrhosis due to the progressive worsening of symptoms as the liver decompensates, potentially leading to painful complications. Unrelieved pain is an important problem as it contributes to impaired sleep, psychological distress, and cognitive dysfunction.^7^^,^^8^ The presence of pain has also been shown to interfere with activities of daily living including physical activities, work, and relationships.^10^^,^^11^ Importantly, unrelieved pain can reduce the quality of living and dying among this growing population.^6^^,^^8^^,^^12^

Recent systematic and scoping reviews indicate that as many as 79%–100% of patients with liver disease report unrelieved pain.^12^, ^13^, ^14^ Common symptoms associated with liver disease include visceral and somatic pain.^12^^,^^15^, ^16^, ^17^ Visceral pain may arise from inflammation of the liver capsule contributing to regional or referred pain, whereas somatic pain includes joint, muscle, and generalized body paim.^6^, ^7^, ^8^^,^^18^ Other pain reports include ascites, muscle cramps, pruritus, and headaches.^6^^,^^7^^,^^18^ Notwithstanding the high prevalence of pain, patients may experience suboptimal pain control due to concerns with impaired drug metabolism, renal dysfunction, and hepatic encephalopathy. These issues may preclude the use of analgesics such as opioids and non-steroidal anti-inflammatory drugs.^12^^,^^13^^,^^19^ As a result of these restrictions, patients may experience unmanaged pain up to the time of death.

Given the lack of research exploring pain in this population, including the relationship between the severity of liver disease and pain, health-care providers lack robust guidance for pain management. A multidimensional approach has been identified as the most effective model for managing chronic and progressive pain to date.^20^^,^^21^ This model views pain from a biopsychosocial perspective and assumes that as the patient’s physical condition worsens, psychological and social factors may also need to be managed. The model uses physical, psychological, social, cognitive, affective, and behavioral measures to best assess and manage each person’s pain condition. By using appropriate assessments to identify modifiable domains, the patient experiencing chronic pain is far more likely to regain function and/or self-reported improvements in quality of life. The biopsychosocial approach to pain has led to the development of therapeutic and cost-effective interdisciplinary pain management programs.^22^^,^^23^

## Aims

At this time, it is unclear whether cirrhosis is related to the pain intensity experienced by patients. Therefore, examining the relationship between cirrhosis and pain is critical to developing targeted pain interventions. This study incorporated biopsychosocial measures of pain among adult patients with cirrhosis including pain location, intensity, and patient report self-care pain management strategies. We also examined the implications of pain on activities of daily living and report the relationship between cirrhosis and pain intensity. Specifically, this study aimed to answer the following research questions: (1) what is the nature of pain experienced by adult patients with cirrhosis? (2) what is the relationship between severity of cirrhosis and pain intensity? (3) how does pain interfere with a patient’s emotional status and activities of daily living? and (4) what self-care management strategies do patients employ when experiencing pain?

## Patients and Methods

### Design

A descriptive cross-sectional survey design was used to explore a biopsychosocial model of the relationship between pain intensity and cirrhosis in a convenience sample of patients.

### Setting

The study was conducted at an ambulatory liver clinic in Toronto General Hospital, Canada.

### Participants

Considering that the study occurred during the third wave of the COVID-19 pandemic between May and September 2021, we followed the hospital’s guidelines for patient recruitment and participation in research studies. The sample size of 118 patients was determined using G∗Power software to achieve 80% power to detect a medium effect size (f^2^ = 0.15) in a multiple linear regression model examining pain intensity’s relationship with the Model for End-stage Liver Disease with Sodium (MELD-Na) scores, with an alpha of 0.05 and up to 10 predictor variables.^24^^,^^25^ As an exploratory study, this sample size was powered to detect a medium effect size, providing a reasonable threshold for detecting meaningful effects and generating important data for powering future studies.

Marketing strategies included informational flyers, invitational letters, and advertisement posters throughout the hospital. The health-care provider introduced the study to eligible patients. Interested patients connected with a member of the research team either by telephone or email to discuss the study. Inclusion criteria required patients to be over 18 years of age, known MELD-Na score within 30 days of the survey, and the ability to read and communicate in English due to resource limitations. Patients with a known diagnosis of cancer (eg, hepatoma) or organ transplantation history (eg, liver, kidney) were excluded since the disease progression and pain experience are known to be different. Informed consent was obtained from eligible patients either by telephone, virtually, or in-person. Consenting patients were offered 3 options for participation: online survey, paper-based mailed survey, or in-person paper-based survey. To mitigate missing values, we employed larger font size that is easy to read and ensured consistency by utilizing the same individual for data collection.^26^^,^^27^

### Measures

The Research Electronic Data Capture, a secure online data management tool, was used to host the survey. This survey was comprised of demographic data (age, sex, education, employment, and income) and the Brief Pain Inventory short form (BPI-SF) to measure pain location, pain intensity, pain interference, and pain management strategies. Severity of liver disease was determined by MELD-Na scores obtained from patient charts within 30 days of survey completion, and biopsychosocial health information was collected from the survey to identify factors that can influence pain.

#### Sociodemographic variables

The sociodemographic variables were reported as categorical variables collected through self-report in the survey. Age was recorded as a continuous variable, while sex, education, marital status, employment, and income were recorded as categorical variables. Sex was dichotomized as male (1) or female (0), and education was categorized as high school or less (1) and at least some college or university (0). Marital status was categorized as being married or have a significant other (2), identify as single (1), and indicating as widowed or divorced or separated (0). Employment status was divided into not able to work (1), retired (0), and working full-time or part-time (0), while income was categorized into 4 levels: <$40,000 (1), $40,000‒$49,999 (2), $50,000‒$79,999 (3), and >$80,000 (4), with the option to select ‘prefer not to say’ (5), a coding scheme chosen to reflect the income distribution of the study population while balancing granularity with participant privacy preferences consistent with common survey practices. These variables are important for understanding the potential influences of sociodemographic factors on pain and related health outcomes. Sociodemographic factors such as education and income can impact access to health-care resources and overall well-being, which in turn may influence pain perception and management strategies.^28^

#### BPI-SF

The BPI-SF^29^ was used as a single administration to measure pain. The BPI-SF has strong reliability and validity psychometrics for measuring sensory pain and multidimensional implications of pain and is recommended in patients with progressive conditions.^30^^,^^31^ Patients were asked to mark their pain location(s) on anterior/posterior body diagrams and to describe the types of pain medications or treatments they were using, and the amount of relief provided by those medications or treatments. Pain intensity was measured on a 0–10 Likert scale, with specific clinical cutoffs used to categorize pain intensity: 0 (no pain), 1–4 (mild pain), 5–6 (moderate pain), and 7–10 (severe pain).^32^ Using 4 dimensions: pain “right now”, “on average”, “at its worst in the last 24 hours”, and “at its least in the last 24 hours”. Pain interference measures the implications of pain on the patient’s activities of daily living using a 0–10 Likert scale using 7 dimensions: “General Activity”, “Mood”, “Walking Ability”, “Normal Work”, “Relations with other people”, “Sleep”, “Enjoyment of life”.

#### Severity of cirrhosis

The MELD-Na is an objective measure to determine the severity of cirrhosis and to prioritize patients on a transplant waiting list.^33^^,^^34^ Scores range from 6 to 40, higher MELD-Na scores predict higher mortality. MELD-Na scores of <17 had <2% 90-day mortality risk, 17–20 had 3%–4% 90-day mortality risk, 21–22 had 7%–10% mortality risk, and 23–31 >14%‒15% mortality risk. MELD-Na scores were obtained from the chart within 30 days of survey completion.

#### Biopsychosocial factors of pain

Physical factors were reported as dichotomous variables of ascites, edema, muscle wasting, and physical comorbidity (Yes = 1/No = 0) reported by the patient in the survey. Ascites, edema, and muscle wasting are common physiological complications that have been reported to influence pain for patients with decompensated cirrhosis.^6^, ^7^, ^8^^,^^12^ Physiological diseases are known to engage the nervous system (nociception) that can stimulate pain receptors.^28^ Psychological factors were reported as dichotomous variables of depression and anxiety (Yes = 1/No = 0). Depression and anxiety are known to amplify pain intensity and have been reported amongst patients with liver disease.^18^^,^^28^ Sociocultural factor, reflecting the influence of the social environment on pain within the biopsychosocial model, was assessed as a dichotomous variable of social support (Yes = 1/No = 0), indicating the presence or absence of supportive relationships. Those who selected *‘No social contact’* were considered having no social support. Social support can influence the patient’s perception and expectations of pain.^28^

#### Self-care pain management strategies

Self-care pain management strategies were reported as open-ended questions. Participating patients listed any treatments or medications they were utilizing for pain. The patients would then identify on an 11-point Likert scale how much relief they experienced in the last 24 hours based on the identified treatment or medication (0% [no relief] to 100% [complete relief]).

### Data Analyses

Sample demographics and item scores were analyzed using descriptive statistics (mean, standard deviation [SD], and frequency distributions). The relationship between pain intensity and MELD-Na was examined using multiple linear regression. Model variables were selected *a priori* as guided by the biopsychosocial model and included: MELD-Na score, physical variables (ascites, edema, muscle wasting, and physical comorbidity), psychological variables (depression and anxiety), and sociocultural (social support). Inspection of the data suggested MELD-Na scores were not linearly related to pain intensity, prompting categorization of MELD-Na scores into 90-day mortality risk categories; these were then modeled using 3 dummy variables (17–20, 21–22, and 23–31), with the reference category (9–16) chosen as the largest sample size.^26^ All explanatory variables were entered simultaneously into the model and retained regardless of *P* value to preserve the biopsychosocial framework’s integrity, with model fit assessed using the F-value for overall significance and adjusted R-squared for explained variance. Therefore, MELD-Na scores were categorized according to the 90-day mortality risk categories and then modelled using 3 dummy variables (17–20, 21–22, and 23–31) with the reference category represented by the category with the largest sample size (9–16). Various diagnostics were conducted to ensure model assumptions were met for homoscedasticity, linearity, bivariate correlation, and multicollinearity.^26^^,^^27^^,^^35^ Statistical analyses were performed using Statistical Package for the Social Sciences (version 26). There was no missing data on any variable.

## Results

### Patient Characteristics

A total of 118 patients with cirrhosis participated in the study. Two other patients (98% response rate) were invited but declined to participate for personal reasons. Table 1 describes the characteristics of the sample. The mean age was 59 years (SD = 11.8) and most patients were male (61.9%). The majority reported partial or complete high school level education (88%), <$40,000 annual household income (61%), and being retired or not able to work (72%). Common physical symptoms of cirrhosis included ascites (89.8%), edema (80.5%), muscle wasting (77.1%), and physical co-morbidity (28.8%). Self-reported psychological conditions comprised depression (13.6%) and anxiety (14.4%). More than half of the patients were married or had a significant other (64.4%) and reported social support outside the home (50.8%).

**Table 1:** Patient Characteristics by Severity of Liver Disease Using the MELD-Na Score

Characteristics	MELD-Na score				
	All (n = 118)	9–16 (n = 69)	17–20 (n = 18)	21–22 (n = 12)	23–31 (n = 19)
Scores below indicate: mean ± SD (range)					
Demographic data					
Age	59 ± 11.8 (21–85)	59 ± 12.9 (21–85)	59 ± 8.4 (41–72)	60 ± 7.6 (56–74)	55 ± 2.6 (34–76)
Scores below indicate: frequency (percentage)					
Sex					
Male	73 (61.9%)	38 (55.1%)	11 (61.1%)	11 (91.7%)	13 (68.4%)
Female	45 (38.1%)	31 (44.9%)	7 (38.9%)	1 (8.3%)	6 (31.6%)
Education					
High school or less	104 (88.1%)	53 (91.3%)	14 (77.8%)	11 (91.7%)	16 (84.2%)
At least some college or university	14 (11.9%)	6 (8.7%)	4 (22.2%)	1 (8.3%)	3 (15.8%)
Employment					
Not able to work	49 (41.5%)	24 (34.8%)	10 (55.5%)	7 (58.3%)	8 (42.1%)
Retired	36 (30.5%)	22 (31.9%)	5 (27.8%)	5 (41.7%)	4 (21.1%)
Working full or part-time	33 (28%)	23 (33.3%)	3 (16.7%)	0	7 (36.8%)
Yearly income					
<$40,000	72 (61%)	39 (56.6%)	12 (66.6%)	9 (75%)	12 (63.2%)
$40,000–$49,999	11 (9.3%)	7 (10.1%)	1 (5.6%)	1 (8.3%)	2 (10.5%)
$50,000–$79,999	18 (15.3%)	13 (18.8%)	1 (5.6%)	1 (8.3%)	3 (15.8%)
>$80,000	16 (13.6%)	10 (14.5%)	4 (22.2%)	0	2 (10.5%)
Prefer not to say	1 (0.8%)	0	0	1 (8.3%)	0
Biopsychosocial clinical features					
Decompensated cirrhosis	106 (89.8%)	58 (84.1%)	18 (100%)	12 (100%)	18 (94.7%)
Primary liver disease					
Viral hepatitis	25 (21.2%)	11 (16%)	4 (22.2%)	2 (16.7)	8 (42.1%)
Alcohol related	41 (34.7%)	21 (30.4%)	4 (22.2%)	7 (58.3%)	9 (47.3%)
Metabolic dysfunction associated steatotic liver disease (MASLD)	10 (8.5%)	6 (8.7%)	1 (5.6%)	2 (16.7%)	1 (5.3%)
Metabolic dysfunction associated steatohepatitis (MASH)	16 (13.5%)	8 (11.6%)	7 (38.8%)	1 (8.3%)	0
Biopsychosocial clinical features					
Primary biliary cirrhosis (PBC)	5 (4.2%)	5 (7.2%)	0	0	0
Primary sclerosing cholangitis (PSC)	7 (5.9%)	6 (8.7%)	0	0	1 (5.3%)
Autoimmune	6 (5.1%)	5 (7.2%)	1 (5.6%)	0	0
Unknown cause	8 (6.8%)	7 (10.2%)	1 (5.6%)	0	0
Physical comorbidity	34 (28.8%)	21 (30.4%)	5 (27.8%)	6 (50%)	2 (10.5%)
Ascites	106 (89.8%)	58 (84.1%)	18 (100%)	12 (100%)	18 (94.7%)
Edema	95 (80.5%)	48 (69.6%)	18 (100%)	10 (83.3%)	19 (100%)
Muscle wasting	91 (77.1%)	45 (65.2%)	16 (88.9%)	12 (100%)	18 (94.7%)
Depression	16 (13.6%)	11 (15.9%)	1 (5.6%)	1 (8.3%)	3 (15.8%)
Anxiety	17 (14.4%)	11 (15.9%)	0	2 (16.7%)	4 (21.1%)
Sociocultural status					
Married or have a significant other	76 (64.4%)	44 (63.8%)	13 (72.2%)	7 (58.3%)	12 (63.2%)
Single	28 (23.7%)	17 (24.6%)	3 (16.7%)	3 (25%)	5 (26.3%)
Widowed/divorced/separated	14 (11.9%)	8 (11.6%)	2 (11.1%)	2 (16.7%)	2 (10.5%)
Social support	60 (50.8%)	33 (47.8%)	11 (61.1%)	6 (50%)	10 (52.6%)
Socialize with family or friends					
Infrequent	58 (49.2%)	36 (52.5%)	7 (38.9%)	6 (50%)	9 (47.4%)
Once a week	13 (11%)	7 (10.1%)	1 (5.6%)	2 (16.7%)	3 (15.8%)
Twice a week	8 (6.85)	5 (7.2%)	2 (11.1%)	0	1 (5.3%)
Three times a week	11 (9.3%)	4 (5.8%)	4 (22.2%)	1 (8.3%)	2 (10.5%)
Four times a week	4 (3.4%)	3 (4.3%)	1 (5.6%)	0	0
Five times a week	8 (6.8%)	5 (7.2%)	1 (5.6%)	0	2 (10.5%)
Six times a week	2 (1.7%)	1 (1.4%)	0	1 (8.3%)	0
Seven times a week	14 (11.9%)	8 (11.6%)	2 (11.1%)	2 (16.7%)	2 (10.5%)

SD, standard deviation.

### Pain Characteristics

#### Pain location

Over 86% (n = 102) of the patients reported pain at the time of survey. Table 2 describes the pain location of the sample. Pain was most often reported in the abdomen (83.1%), lower legs (59.3%), and lower back (56.8%). Locations associated with the highest pain intensity were the abdomen (72.9%), lower back (36.4%), and lower legs (33.9%).

**Table 2:** Pain Location

Characteristics	MELD-Na score				
	All (n = 118)	9–16 (n = 69)	17–20 (n = 18)	21–22 (n = 12)	23–31 (n = 19)
Reported pain location					
Head	9 (7.6%)	3 (4.3%)	3 (16.7%)	1 (8.3%)	2 (10.5%)
Face	2 (1.7%)	1 (1.4%)	1 (5.6%)	0	0
Neck	17 (14.4%)	7 (10.2%)	5 (27.8%)	1 (8.3%)	4 (21.2%)
Shoulder	8 (6.8%)	4 (5.8%)	2 (11.1%)	1 (8.3%)	1 (5.3%)
Upper arms	3 (2.5%)	1 (1.4%)	1 (5.6%)	0	1 (5.3%)
Lower arms	5 (4.2%)	3 (4.3%)	1 (5.6%)	0	1 (5.3%)
Chest	11 (9.3%)	7 (10.1%)	1 (5.6%)	0	3 (15.8%)
Abdomen/stomach	98 (83.1%)	50 (72.5%)	17 (94.4%)	12 (100%)	19 (100%)
Perineal	22 (18.6%)	13 (11%)	4 (22.2%)	1 (8.3%)	4 (21.2%)
Upper legs	45 (38.1%)	23 (33.3%)	6 (33.3%)	6 (50%)	10 (52.6%)
Lower legs	70 (59.3%)	36 (52.2%)	11 (61.1%)	8 (66.7%)	15 (78.9%)
Back of head	0	0	0	0	0
Upper back	5 (4.2%)	2 (2.9%)	1 (5.6%)	0	2 (10.5%)
Mid back	10 (8.5%)	5 (7.2%)	1 (5.6%)	0	4 (21.2%)
Lower back	67 (56.8%)	38 (55.1%)	10 (55.6%)	8 (66.7%)	11 (57.9%)
Buttocks	3 (2.5%)	1 (1.4%)	2 (11.1%)	0	0
Reported pain that hurts the most					
Head	1 (0.8%)	0	0	0	1 (5.3%)
Face	0	0	0	0	0
Neck	4 (3.4%)	1 (1.4%)	0	1 (8.3%)	2 (10.5%)
Shoulder	0	0	0	0	0
Upper arms	1 (0.8%)	0	0	1 (8.3%)	0
Lower arms	3 (2.5%)	2 (2.9%)	1 (5.6%)	0	0
Chest	4 (3.4%)	2 (2.9%)	0	0	2 (10.5%)
Abdomen/stomach	86 (72.9%)	48 (69.6%)	13 (72%)	10 (83.3%)	15 (78.9%)
Perineal	9 (7.6%)	5 (7.2%)	1 (5.6%)	0	3 (15.8%)
Upper legs	23 (19.5%)	14 (20.3%)	2 (11.1%)	3 (25%)	4 (21.2%)
Lower legs	40 (33.9%)	23 (33.3%)	3 (16.7%)	6 (50%)	8 (42.1%)
Back of head	0	0	0	0	0
Upper back	2 (1.7%)	1 (1.4%)	0	0	1 (5.3%)
Mid back	4 (3.45)	2 (2.9%)	0	0	2 (10.5%)
Lower back	43 (36.4%)	25 (36.2%)	8 (44.4%)	5 (41.7%)	5 (26.3%)
Buttocks	1 (0.8%)	1 (1.4%)	0	0	0

#### Pain intensity

Pain intensity at the time of survey completion (ie, right now) had a mean score of 4.1 (mild pain) (SD = 2.9) out of 10. Pain average had a mean of 3.5 (mild pain) (SD = 2.8) out of 10. Pain at its worst in the last 24 hours had a mean of 6 (moderate pain) (SD = 2.9) out of 10. Pain at its least in the last 24 hours had a mean of 2.2 (mild pain) (SD = 2.2) out of 10. The composite score of the 4 pain dimensions had a mean of 4 (mild pain) (SD = 2.4) out of 10 (Table 3).

**Table 3:** Pain Intensity

Pain intensity (out of 10)	Pain right now n (%)	Pain average n (%)	Pain at its worst in the last 24 h n (%)	Pain at its least in the last 24 h n (%)
0	16 (13.6%)	28 (23.7%)	3 (2.5%)	38 (32.2%)
1	12 (10.2%)	10 (8.5%)	4 (3.4%)	18 (15.3%)
2	10 (8.5%)	9 (7.6%)	11 (9.3%)	14 (11.9%)
3	15 (12.7%)	9 (7.6%)	15 (12.7%)	14 (11.9%)
4	14 (11.9%)	13 (11%)	4 (3.4%)	14 (11.9%)
5	13 (11%)	18 (15.3%)	13 (11%)	11 (9.3%)
6	9 (7.6%)	9 (7.6%)	8 (6.8%)	4 (3.4%)
7	9 (7.6%)	14 (11.9%)	17 (14.4%)	2 (1.7%)
8	11 (9.3%)	7 (5.9%)	13 (11%)	3 (2.5%)
9	6 (5.1%)	0	15 (12.7%)	0
10	3 (2.5%)	1 (0.8%)	15 (12.7%)	0
Scores below indicate: mean, standard deviation (range)				
Pain intensity (out of 10)				
Pain right now	4.1 ± 2.9 (0–10)			
Pain average	3.5 ± 2.8 (0–10)			
Pain at its worst in the last 24 h	6 ± 2.9 (0–10)			
Pain at its least in the last 24 h	2.2 ± 2.2 (0–8)			
Composite mean score	4 ± 2.4 (0–8.5)			

#### Pain interference

The highest average pain interference score was reported in the domain of general activity (5.8, SD = 3, range = 0–10) followed by walking ability (5.7, SD = 3.3, range = 0–10). These were followed by the pain interference scores of sleep at 5.6 (SD = 3.6, range = 0–10) and Enjoyment of life at 4.6 (SD = 3.8, range = 0–10). The composite score of the 7 dimensions of pain interference was 4.8 (SD = 3, range = 0–10) (Table 4).

**Table 4:** Pain Interference

Pain interference (out of 10)	General activity n (%)	Mood n (%)	Walking ability n (%)	Normal work n (%)	Relationship with others n (%)	Sleep n (%)	Enjoyment of life n (%)
0 (does not interfere)	5 (4.2%)	38 (32.2%)	8 (6.8%)	14 (11.9%)	40 (33.9%)	16 (13.6%)	34 (28.8%)
1	7 (5.9%)	14 (11.9%)	7 (5.9%)	7 (5.9%)	12 (10.2%)	7 (5.9%)	8 (6.8%)
2	12 (10.2%)	4 (3.4%)	12 (10.2%)	10 (8.5%)	7 (5.9%)	6 (5.1%)	2 (1.7%)
3	10 (8.5%)	6 (5.1%)	12 (10.2%)	10 (8.5%)	12 (10.2%)	10 (8.5%)	9 (7.6%)
4	7 (5.9%)	5 (4.2%)	6 (5.1%)	6 (5.1%)	7 (5.9%)	6 (5.1%)	3 (2.5%)
5	14 (11.9%)	12 (10.2%)	8 (6.8%)	13 (11%)	12 (10.2%)	12 (10.2%)	7 (5.9%)
6	9 (7.6%)	6 (5.1%)	11 (9.3%)	7 (5.9%)	4 (3.4%)	8 (6.8%)	7 (5.9%)
7	13 (11%)	12 (10.2%)	10 (8.5%)	14 (11.9%)	9 (7.6%)	10 (8.5%)	12 (10.2%)
8	10 (8.5%)	8 (6.8%)	9 (7.6%)	9 (7.6%)	5 (4.2%)	6 (5.1%)	9 (7.6%)
9	14 (11.9%)	1 (0.8%)	13 (11%)	14 (11.9%)	3 (2.5%)	6 (5.1%)	11 (9.3%)
10 (completely interferes)	17 (14.4%)	12 (10.2%)	22 (18.6%)	14 (11.9%)	6 (5.1%)	31 (26.3%)	16 (13.6%)
Scores below indicate: mean, standard deviation (range)							
Pain interference (out of 10)							
General activity	5.8 ± 3.1 (0–10)						
Mood	3.7 ± 3.6 (0–10)						
Walking ability	5.7 ± 3.3 (0–10)						
Normal work	5.3 ± 3.3 (0–10)						
Relationship with others	3.1 ± 3.2 (0–10)						
Sleep	5.6 ± 3.6 (0–10)						
Enjoyment of life	4.6 ± 3.8 (0–10)						

### Self-Care Pain Management Strategies

Table 5 outlines the self-care pain management strategies. The majority of patients (62.7%) did not report using any medications or other treatments for their pain. Only 17.8% of the patients reported having greater than 50% pain relief from their pain management strategies. The most common reported pharmacological pain management strategy was acetaminophen (27.1%) and the only nonpharmacological strategy reported was physical positioning (0.8%).

**Table 5:** Self-Care Pain Management Strategies

Pain management	All (n = 118)
Pharmacological strategies	
Acetaminophen/tylenol	32 (27.4%)
Advil	1 (0.8%)
Aspirin	1 (0.8%)
Gabapentin	1 (0.8%)
Hydromorphone	5 (4.2%)
Nonspecified opioid	1 (0.8%)
None	74 (62.7%)
Percocet	2 (1.7%)
Nonpharmacological strategies	
Reclining position to allow pressure relief	1 (0.8%)

### Relationship Between Severity of Cirrhosis and Pain Intensity

Table 6 presents the results of the multiple linear regression of the composite mean score representing pain intensity on the biopsychosocial factors (MELD-Na 17–20, MELD-Na 21–22, MELD-Na 23–31, Physical comorbidity, Ascites, Edema, Muscle Wasting, Depression, Anxiety, and Social support). Overall, the model explained over 50% of the variance in pain intensity (adjusted R Square = 0.52, *P* < .001). The model shows that the severity of cirrhosis as indicated by the MELD-Na score categories is independently associated with pain intensity. The MELD-Na scores of 23‒31, compared to reference group of MELD-Na of 9‒16, had the largest effect on pain intensity of the severity categories (β = 0.43, 95% confidence interval [CI; 1.86–3.74], *P* < .001), followed by each of MELD-Na scores of 21‒22 (β = 0.28, 95% CI [1.17–3.35], *P* < .001), and MELD-Na 17–20 (β = 0.19, 95% CI [0.34–2.25], *P* = .008) compared to the reference group. Of the other biopsychosocial variables, included presence of ascites (β = 0.16, 95% CI [0.03–2.59], *P* = .046) and edema (β = 0.28, 95% CI [0.8–2.66], *P* < .001) were both significantly associated with the composite pain intensity mean score. The variables depression, anxiety, and social support in the model were not statistically significant in explaining the variance of pain intensity.

**Table 6:** Biopsychosocial Variables in the Multiple Linear Regression for Composite Pain Intensity

Variable	Standardized beta coefficients (95% CI)	Significance
MELD-Na 17–20	0.19 (0.34–2.25)	0.008
MELD-Na 21–22	0.28 (1.17–3.35)	<0.001
MELD-Na 23–31	0.43 (1.86–3.74)	<0.001
Physical comorbidity	0.13 (−0.05 to 1.43)	0.067
Ascites	0.16 (0.03–2.59)	0.046
Edema	0.28 (0.8–2.66)	<0.001
Muscle wasting	0.09 (−0.51 to 1.56)	0.318
Depression	0.03 (−0.81 to 1.29)	0.654
Anxiety	0.08 (−0.47 to 1.55)	0.289
Social support	0.08 (−0.26 to 1.02)	0.240

(Reference group MELD-Na score 9–16). Dependent variable: Composite pain intensity mean score. Explanatory multiple variables entered simultaneously: MELD-Na (3 Dummy variables: MELD-Na score 17–20, MELDs-Na score 21–22, MELD-Na score 23–31), Physical comorbidity, Ascites, Edema, Muscle wasting, Depression, Anxiety, and Social support.

MELD-Na, Model for End-stage Liver Disease with Sodium.

## Discussion

This cross-sectional survey study examined the relationship between the biopsychosocial factors of pain and pain intensity among adult patients in Canada with cirrhosis. Most patients reported moderate to severe pain intensity in the last 24-hours. Disease symptoms including ascites and edema were associated with pain intensity. Pain interfered with general activities, walking, sleeping, and enjoyment of life. Most reported not taking analgesia; those taking analgesia experienced incomplete pain relief. Our regression model suggests that higher MELD-Na scores have a stronger relationship with pain intensity when compared to lower MELD-Na scores.

Our findings align with studies reporting the presence of unmanaged pain in patients with cirrhosis.^12^^,^^14^^,^^17^ For example, ambulatory patients with end-stage liver disease (eg, MELD score ≥18) in Hansen et al’s^36^ study reported moderate to severe abdominal and back pain. In our study, patients using analgesia reported less than 50 percent relief of their pain symptoms. As the severity of cirrhosis progresses, the patient may experience physiological changes that can affect their risk of painful musculoskeletal complications. For instance, portal vein hypertension can lead to fluid retention (ascites and edema), parietal wall varices, abdominal wall hernia, metabolic bone disease, arthritis, and osteomyelitis. Our findings indicate that ascites and edema were the most commonly reported symptoms of cirrhosis and may lead to physical pain in the abdomen, joints, and back. This suggests that the relationship between severity of cirrhosis and pain intensity may be related to the physical implications of the disease process.

The study focused on different types of pain such as acute, chronic, visceral, somatic, and liver-specific pain. Visceral pain typically stemmed from liver-related complications, whereas somatic pain included musculoskeletal and skin-related discomfort. Somatic pain included joint and back pain, which can be related to physical deconditioning or musculoskeletal changes.^7^ Episodic events such as ascites can be acute related pain requiring immediate and effective management to prevent further complications.^8^ Acute pain necessitates a nuanced approach to pain management that addresses the underlying liver disease while managing the pain symptoms effectively. Persistent pain, on the other hand, can be chronic and a constant presence that impacts the patients' quality of life and require ongoing management strategies such as back pain.^37^ These various types of pain highlight the complexity of pain management in patients with cirrhosis and the need for comprehensive pain management strategies that consider the multidimensional nature of pain.

In contrast to a prior study by Hansen et al,^36^ we identified a relationship between severity of cirrhosis and pain intensity. A possible explanation for this difference is our larger sample size^36^ and our sampling distribution of patients across several levels of disease severity that may better represent the breadth of the pain experience for patients with cirrhosis. Evaluation of severity of liver disease has been shown to be an effective approach to the selection and/or scheduling of medical treatment.^38^^,^^39^ The recent American Association for the Study of Liver Diseases Practice Guidance^19^ recommends routine appraisal of symptom severity for patients with decompensated cirrhosis. Progression in the severity of liver disease could act as a prompt to conduct a comprehensive pain assessment.

We noted fewer pain self-care management strategies in our study population than is reported in another study cohort.^36^ However, the level of pain relief in response to analgesia is similarly low. For example, Madan and colleagues^40^ found that patients with cirrhosis experienced limited analgesic effect and only 33% reported pain relief on average. The lack of reported pain relief suggests that patients may not be utilizing their pain medication optimally or such treatment is ineffective. Metabolism of pain medications such as opioids is theorized to be reduced because of declining liver function.^6^^,^^7^^,^^19^ Health-care providers may be challenged with limited effective pharmacological options and concern of hepatic encephalopathy with increasing analgesic dosage. Limited use of pharmacological and nonpharmacological pain management strategies in our study participants may explain the average “pain at its worst in the last 24 hours” moderate to severe pain intensity scores. Notably, 62.7% of participants did not report using any pharmacological pain treatments, yet pain interference scores indicate moderate disruption to daily activities such as general activity (5.8 ± 3.1), walking ability (5.7 ± 3.3), and sleep (5.6 ± 3.6). This suggests that the absence of medication use does not necessarily reflect minimal pain impact, but rather potential barriers to effective pain management amongst this population, such as restricted analgesic options and under reporting, which merits further investigation. Comparisons with recent studies highlight these challenges. Rubin et al^41^ reported that among 5333 patients with cirrhosis including 39% transplant candidates, 60% with significant pain used analgesics (34% opioids), yet pain persisted increasing hospital utilization (adjusted odds ratio 1.3, *P* < .001) and mortality (adjusted hazard ratio 1.4, *P* < .001. In contrast, our nontransplant participant’s lower analgesic use (37.3%, mostly acetaminophen) suggests greater under treatment, possibly reflecting reluctance to use opioids in ambulatory settings. Shapuram et al^42^ found that in 459 patients with acute on chronic liver failure and decompensated cirrhosis, including 30 transplant recipients, poor emotional well-being and energy predicted mortality (adjusted hazard ratio 0.93 and 0.91, *P* < .05), suggesting that psychological distress may exacerbate pain perception and complicate effective pain management in this population. This highlights the need for integrated psychological support in pain management strategies, as emotional well-being can influence pain outcomes.^28^ In our study, the burden of persistent pain in nontransplant patients highlights unmet needs in this cohort. In addition, despite abdominal pain being the most prevalent site (83.1%), no participants reported using proton pump inhibitors, a common self-medication strategy in clinical practice for gastrointestinal symptoms. This absence may reflect under reporting, a focus on analgesics in our survey design or participants not associating proton pump inhibitors with pain relief, suggesting a need for future studies to explicitly examine gastrointestinal symptom management. Patients rely on the recommendations of health-care providers to treat pain. Pharmacological strategies alone may be inadequate to treat pain in complex and progressive health conditions such as cirrhosis. Consideration of nonpharmacological pain strategies may be important in achieving improved pain outcomes in this population.^19^^,^^20^ Current evidence suggests that pain is a multidimensional construct and thus warrants a multidimensional treatment plan.^19^, ^20^, ^21^

While our analyses did not show a relationship between psychological and social factors of pain with pain intensity, there is strong evidence in other studies to suggest otherwise in other disease conditions such as hepatitis C^47^. Psychological factors that have an influence on mood and emotional status have been reported to be predictors of pain in patients with liver disease.^17^ This is consistent in other populations where depression and anxiety had an independent relationship with pain intensity.^43^ Our binary depression/anxiety measures may have missed nuanced psychological contributions reflecting our study’s motivation of the need for short assessments to reduce the study burden and facilitate simplicity, where the patients had limited time and cognitive resources to engage in lengthy assessments. Single-item questions are known to have limitations in terms of validity and reliability.^44^ While they offer convenience and ease of administration, they may not capture the full complexity of psychological constructs such as depression and anxiety. Multi-item scales, such as the Patient Health Questionnaire for depression and the generalized anxiety disorder for anxiety, provide a more comprehensive assessment and have been extensively validated in various populations.^45^^,^^46^ The use of single-item measures likely reduced sensitivity to subtle psychological variations and oversimplified complex experiences, limiting their validity and analytical power. Psychological conditions like depression and anxiety exist on a spectrum, and capturing this range is crucial for understanding their impact on patients' health outcomes. The work of Sweeney et al^47^ demonstrates the significance of appraising psychological factors in patients with chronic illnesses where depression and anxiety not only exacerbated the pain experience but are worsened by persistent pain creating a vicious cycle that impacts the patient’s quality of life. Reliable and valid appraisal tools are important to accurately determine the presence of those modifiable factors.

The sociocultural domain of the biopsychosocial factor considers the impact of social support on pain perception and management. Although our analyses did not establish a significant relationship between social support and pain intensity, social isolation can exacerbate the pain experience by increasing feelings of loneliness and reducing access to support networks that can provide emotional and practical assistance.^28^ Existing research has shown that social support is a critical determinant of health outcomes in this population. For example, social support has been associated with pain intensity in patients with Hepatitis C^43^. Nearly half of the participants in this study reported limited social interactions. Social support can buffer the impact of pain by providing emotional comfort, practical help, and a sense of belonging.^28^ Multidimensional strategies that address psychological and social factors of pain can have an influence on pain intensity.

Unrelieved pain can significantly affect quality of life.^12^ Our findings indicate a pain prevalence of 86% during the study, which is considered very high according to a recent^13^ scoping review. This high prevalence is significant because pain intensity is known to be associated with pain interference. This can include interference with walking, self-care, employment, and socialization. Furthermore, the presence of pain can significantly impact sleep as observed in our results.^12^^,^^13^ Current evidence indicates that sleep disturbance is associated with increased pain intensity.^48^ Prior epidemiological studies have documented an association between past-month pain interference and mood or major depressive disorders.^49^ Pain is also associated with increased risk for harmful substance use: those with pain conditions who experience poor treatment outcomes are less likely to remain abstinent from alcohol or other drugs.^50^ These findings suggest that providers should consider screening patients with moderate or severe pain interference for mood, anxiety, and substance-use problems and monitor the possible development of subsequent comorbid psychiatric conditions.

During the COVID-19 pandemic, there was a notable increase in the prevalence of mental health issues among Canadians. According to Statistics Canada,^51^ the prevalence of major depressive disorder increase from 15% in 2020 to 19% in 2021, and the prevalence of generalized anxiety disorder rose from 13% to 15% during the same period. These increases highlight the significant psychological impact of the pandemic, particularly in relation to the stressors associated with lockdowns, social isolation, and economic uncertainty.^52^ In our study, the prevalence of self-reported depression and anxiety was reported to be 13.6% and 14.4%, which appear to be lower than reported by Statistics Canada. Our study participants may differ in significant ways from the general Canadian population. Differences in demographics, health status, and social support networks could influence the prevalence of depression and anxiety. However, these differences may also reflect our use of single-item self-report questions to assess depression and anxiety, compared to multi-item validated scales typically used in broader populations. The pandemic also likely affected participants’ ability to access pain management services. Restrictions and reallocation of health-care resources to manage COVID-19 may have limited patients’ access to treatments and support systems. Furthermore, the reduction in social interactions due to lockdowns and social distancing measures could have compounded feelings of isolation and loneliness, further exacerbating psychological distress and pain experiences. Given these contextual factors, our findings must be interpreted with caution. It is crucial to acknowledge that the pandemic created an unusual and highly stressful environment, which may not reflect typical conditions.

Our study addressed common limitations of previous research^13^ such as nonresponse bias, measurement inconsistency, and uncontrolled confounding by implementing specific methodological improvements. The implementation of follow-up contacts effectively minimized nonresponse bias. The use of validated instruments for pain and cirrhosis measures, specifically the BPI-SF and MELD-Na scores, enhanced the reliability and accuracy of our data collection. Several limitations to this study are worth noting. Firstly, our convenience sampling method was from a single site limiting the representativeness of our data. Secondly, the sample size of 118 participants provided sufficient power to explain over 50% of the variance in our regression model (adjusted R^2^ = 0.52) but may be considered small relative to the heterogeneity of cirrhosis, potentially limiting generalizability and the ability to detect small effects of individual predictors. Thirdly, this type of research explores associations, and we cannot infer causality. Fourthly, our data collection was based on a single time collection that may not fully represent the patient’s pain experience. In addition, the inclusion of 69 patients (58.5%) with MELD-Na scores <17, indicative of relatively less severe liver disease (<2% 90-day mortality risk), could potentially skew findings toward milder pain experiences; however, stratified analyses reveal consistent pain prevalence across groups (eg, 72.5% abdominal pain in MELD-Na 9–16 vs 100% MELD-Na 23–31), though pain intensity increases with severity (β = 0.43 for MELD-Na 23–31, *P* < .001) suggesting that the absence of patients with MELD-Na >31 may still underestimate the full spectrum of pain in advanced disease. The timing of the data collection occurred during the COVID-19 pandemic that could have impacted the participants’ experience with depression and anxiety, ability to access pain management, and limit social interactions. The pandemic could have exacerbated psychological distress among patients, thereby affecting their responses and the overall study results. Furthermore, our sample did not include patients with severity of liver disease scores greater than 31 which suggests that the pain experience of patients with MELD-Na scores between 32‒40 require further investigation. Lastly, it is important to acknowledge the lack of validated scales for depression and anxiety in this study. This limitation is primarily due to the high illness burden observed in our study participants, which necessitated making pragmatic choices regarding the selection of study measurement tools. Future research should validate depression and anxiety measurement tools in this population to enhance the accuracy of identifying psychological factors of pain. Inclusion of reliable and valid depression and anxiety measurement tools can enhance the accuracy of identifying the psychological factors of pain.

## Conclusion

In summary, we identified a relationship between severity of liver disease and pain intensity. The high prevalence of pain may be an indication of the lack of effective pain management which can interfere with the patient’s activities of daily living and quality of life. Future research should employ random sampling from multiple sites to rigorously validate the relationship between liver disease severity and pain intensity, enhancing the generalizability of these findings. Given that disease severity strongly predicts pain intensity, with physical factors like ascites and edema also contributing, future studies should employ pain assessment tools that comprehensively evaluate these physical drivers alongside psychological and social factors to identify modifiable targets for both pharmacological and nonpharmacological interventions, addressing the persistent pain and limited relief observed despite varying disease severity.
